# Reciprocal Regulation of AMPK/SNF1 and Protein Acetylation

**DOI:** 10.3390/ijms19113314

**Published:** 2018-10-25

**Authors:** Ales Vancura, Shreya Nagar, Pritpal Kaur, Pengli Bu, Madhura Bhagwat, Ivana Vancurova

**Affiliations:** Department of Biological Sciences, St. John’s University, New York, NY 11439, USA; shreya.nagar17@my.stjohns.edu (S.N.); pritpal.kaur17@my.stjohns.edu (P.K.); bup@stjohns.edu (P.B.); madhura.bhagwat16@my.stjohns.edu (M.B.); vancuroi@stjohns.edu (I.V.)

**Keywords:** AMP-activated protein kinase, epigenetics, protein acetylation, KATs, HDACs, acetyl-CoA, NAD^+^

## Abstract

Adenosine monophosphate (AMP)-activated protein kinase (AMPK) serves as an energy sensor and master regulator of metabolism. In general, AMPK inhibits anabolism to minimize energy consumption and activates catabolism to increase ATP production. One of the mechanisms employed by AMPK to regulate metabolism is protein acetylation. AMPK regulates protein acetylation by at least five distinct mechanisms. First, AMPK phosphorylates and inhibits acetyl-CoA carboxylase (ACC) and thus regulates acetyl-CoA homeostasis. Since acetyl-CoA is a substrate for all lysine acetyltransferases (KATs), AMPK affects the activity of KATs by regulating the cellular level of acetyl-CoA. Second, AMPK activates histone deacetylases (HDACs) sirtuins by increasing the cellular concentration of NAD^+^, a cofactor of sirtuins. Third, AMPK inhibits class I and II HDACs by upregulating hepatic synthesis of α-hydroxybutyrate, a natural inhibitor of HDACs. Fourth, AMPK induces translocation of HDACs 4 and 5 from the nucleus to the cytoplasm and thus increases histone acetylation in the nucleus. Fifth, AMPK directly phosphorylates and downregulates p300 KAT. On the other hand, protein acetylation regulates AMPK activity. Sirtuin SIRT1-mediated deacetylation of liver kinase B1 (LKB1), an upstream kinase of AMPK, activates LKB1 and AMPK. AMPK phosphorylates and inactivates ACC, thus increasing acetyl-CoA level and promoting LKB1 acetylation and inhibition. In yeast cells, acetylation of Sip2p, one of the regulatory β-subunits of the SNF1 complex, results in inhibition of SNF1. This results in activation of ACC and reduced cellular level of acetyl-CoA, which promotes deacetylation of Sip2p and activation of SNF1. Thus, in both yeast and mammalian cells, AMPK/SNF1 regulate protein acetylation and are themselves regulated by protein acetylation.

## 1. AMPK Links Metabolism and Signaling with Protein Acetylation, Epigenetics, and Transcriptional Regulation

AMP-activated protein kinase (AMPK) is highly conserved across eukaryotes and serves as an energy sensor and master regulator of metabolism, functioning as a fuel gauge monitoring systemic and cellular energy status [1,2,3]. Activation of AMPK occurs when the intracellular AMP/ATP ratio increases. In general, AMPK inhibits anabolism to minimize energy consumption and activates catabolism to increase ATP production.

AMPK is a heterotrimeric complex composed of subunit and two regulatory subunits, α and γ. The human genome contains two genes encoding two distinct subunits, 1 and 2, two α subunits, β1 and β2, and three γ subunits, γ1, γ2, and γ3 [1,2,3]. Different combinations of α, β, and γ subunits can produce 12 distinct AMPK complexes; however, it is not known whether these complexes differ in substrate specificities, subcellular localization, or other aspects of regulation. The subunit features the catalytic protein kinase domain, the α subunit contains a carbohydrate-binding domain that allows AMPK to interact with glycogen [4], and the γ subunit contains domains that bind AMP and thus impart AMPK regulation by cellular energy state [5,6,7].

The AMPK complex is activated more than 100-fold by phosphorylation on Thr172 of the catalytic α subunit [8]. The major upstream kinase targeting this site is the tumor suppressor liver kinase B1 (LKB1) [9,10,11]. LKB1 is responsible for most of AMPK activation under low energy conditions in the majority of tissues, including liver and muscle [2,12,13,14]. LKB1 is also responsible for AMPK activation in response to mitochondrial insults [15].

AMPK targets a number of metabolic enzymes and transporters, such as glucose transporter (GLUT) 1 and GLUT4, glycogen synthase (GS), acetyl-CoA carboxylase (ACC), and hydroxymethylglutaryl-CoA reductase (HMGCR) [16,17,18]. AMPK also regulates metabolism at the transcriptional level by phosphorylating sterol regulatory element-binding protein 1 (SREBP1), carbohydrate-responsive element-binding protein (ChREBP), transcriptional coactivator peroxisome proliferator-activated receptor gamma coactivator 1-alpha (PGC1) and transcriptional factor forkhead box O3 (FOXO3) [19,20,21,22].

One of the most important targets of AMPK is mechanistic target of rapamycin complex 1 (mTORC1). mTOR is a conserved serine/threonine protein kinase from the phosphatidylinositol-3-kinase (PI3K) family. mTOR is found in all eukaryotes and forms the catalytic subunit of mTORC1 and mTORC2. mTORC1 is regulated by nutrients and growth factors, and functions as a master regulator of cell growth and metabolism by phosphorylating a host of targets [23]. The AMPK-dependent mechanisms of mTORC1 inhibition are mediated by phosphorylation of the tuberous sclerosis complex (TSC) and raptor subunit of mTORC1 [24,25]. TSC functions as a GTPase activating protein (GAP) for the small GTPase Rheb, which directly binds and activates mTORC1. Thus, by downregulating Rheb, TSC inhibits mTORC1 and downregulation of TSC, which therefore leads to activation of mTORC1. In addition to integrating signals from several growth factor pathways, TSC is also regulated by AMPK. Activated AMPK directly phosphorylates TSC2 on serine residues that are distinct from those regulated by growth factor pathways, resulting in TSC activation and mTORC1 inhibition. In addition to TSC2, AMPK also phosphorylates mTORC1 subunit Raptor, leading again to mTORC1 inhibition [23,25]. Under low energy conditions, AMPK assembles in a complex with v-ATPase, Ragulator, scaffold protein Axin, and LKB1 on the lysosome surface, resulting in AMPK activation. At the same time, mTORC1 dissociates from the Ragulator and lysosome, resulting in mTORC1 inhibition [26,27]. These results further illustrate that AMPK and mTORC1 are inversely regulated and represent a molecular switch between catabolism and anabolism.

This review focuses on the previously little explored role of AMPK in regulation of acetyl-CoA and NAD^+^ homeostasis and on reciprocal regulation of AMPK and protein acetylation, which places AMPK at the interface between metabolism and other essential cellular functions, including transcription, replication, DNA repair, and aging [14,28,29,30].

## 2. Protein Acetylation

Protein acetylation is a posttranslational protein modification in which the acetyl group from acetyl-CoA is transferred onto ε-amino group of lysine residues. Histones were the first proteins known to be acetylated. More recently, genomic and proteomic approaches in bacteria, yeast, and higher eukaryotes identified many non-histone proteins that are acetylated, suggesting that acetylation extends beyond histones. A number of proteomic studies show that acetylation occurs at thousands of sites throughout eukaryotic cells and that the human proteome contains at least ~2500 acetylated proteins [31,32,33,34]. In comparison, similar analyses of human and mouse proteins identified ~2200 phosphoproteins [35,36]. Thus, it appears that protein acetylation is as widespread as phosphorylation [37]. In human cells, acetylated proteins are involved in the regulation of diverse cellular processes, including chromatin remodeling, the cell cycle, RNA metabolism, cytoskeleton dynamics, membrane trafficking, and key metabolic pathways, such as glycolysis, gluconeogenesis, and the citric acid cycle [32,34]. In general, protein acetylation can both activate and inhibit enzymatic activity of proteins as well as interactions between proteins [28,29,38,39]. Acetylation of histones affects the chromatin structure and transcriptional regulation by two mechanisms. It neutralizes positive charges of lysines and thus diminishes interaction of histone tails with DNA. By forming acetyllysines, histone acetylation creates sites that are recognized and bound by proteins and protein complexes that contain bromodomains. Many of these bromodomain-containing protein complexes covalently or noncovalently modify chromatin structure and thus regulate transcription [40,41].

### 2.1. KATs and HDACs

The enzymes that catalyze protein acetylation were originally called histone acetyltransferases (HATs) [40]. With the realization that histones are not the only substrates, these enzymes are now more commonly referred to as lysine acetyltransferases (KATs) [41,42,43,44,45]. The human genome contains 22 genes that encode proteins currently known to possess protein acetyltransferase activity [39]. The KATs can be classified into three major groups: The GNAT, MYST, and p300/CBP families. Most KATs are catalytic subunits of multiprotein complexes; the noncatalytic subunits of these complexes are typically responsible for substrate recognition, regulation, and subcellular localization. 

Protein acetylation is a dynamic modification. The acetyl groups are removed from proteins by histone deacetylases (HDACs), sometimes also called lysine deacetylases (KDACs) to indicate that acetylated histones are not the only substrates [39]. However, the name HDACs is still more commonly used. HDACs hydrolyze the amide linkage between the acetyl group and amino group of lysine residues, yielding acetate. HDACs are grouped into four classes. Class I, II, and IV are Zn^2+^-dependent amidohydrolases, while class III uses NAD^+^ as a cosubstrate [46]. Class III HDACs are known as the sirtuins [47].

The dynamic balance between protein acetylation and deacetylation, mediated by the activities of KATs and HDACs, is well regulated in healthy cells, but is often dysregulated in cancer and other pathologic conditions. For example, change in the acetylation status of chromatin histones alters the structure of chromatin and expression pattern of genes in cancer cells [43].

### 2.2. Nonenzymatic Acetylation of Mitochondrial Proteins

The reactivity of metabolites depends on the presence of nucleophilic or electrophilic groups. The carbonyl group is electrophilic and can be further enzymatically activated by adding electronegative groups, such as thiols or phosphates. Compounds containing a reactive thioester group in the form of CoA are common in many metabolic pathways and reactions, involving fatty acid synthesis, tricarboxylic acid cycle, amino acid metabolism, and protein acetylation [48]. Protein acetylation by KATs employs a common catalytic mechanism which involves the formation of a ternary complex of KAT-acetyl-CoA-histone and the deprotonation of the ε-amino group of lysine by a glutamate or aspartate residue within the active site of a KAT, followed by a nucleophilic attack on the carbonyl group of acetyl-CoA [42]. 

High concentration of acetyl-CoA coupled with high pH, conditions that exist in the mitochondrial matrix, create a permissive environment for non-enzymatic acetylation of proteins [49,50]. In *Saccharomyces cerevisiae*, about 4000 lysine acetylation sites were identified, many of them on mitochondrial proteins [49,51]. The acetylation of mitochondrial proteins correlates with acetyl-CoA levels in mitochondria, as demonstrated by the fact that acetylation of mitochondrial proteins is dependent on *PDA1*, encoding a subunit of the pyruvate dehydrogenase (PDH) complex [49]. The acetylation of mitochondrial proteins was also elevated by introducing the *cit1*∆ mutation. *CIT1* encodes mitochondrial citrate synthase; *cit1*∆ mutants are not able to utilize acetyl-CoA for citrate synthesis and probably have an elevated level of mitochondrial acetyl-CoA. These results suggest that most of the mitochondrial acetyl-CoA in exponentially growing cells is derived from glycolytically-produced pyruvate that was translocated into mitochondria and converted to acetyl-CoA by the PDH complex. Inactivation of the PDH complex results in about a 30% decrease in cellular acetyl-CoA; this indicates that mitochondrial acetyl-CoA represents about 30% of the cellular pool. However, since mitochondria occupy only 1–2% of the cellular volume in *S. cerevisiae* [52], the mitochondrial acetyl-CoA concentration is about 20–30-fold higher than the concentration in the nucleocytosolic compartment and is probably within the millimolar range [49,50,53,54]. Due to the extrusion of protons across the inner mitochondrial membrane, the pH of the mitochondrial matrix is higher than the pH in the cytosol or nucleus, about 8.0 [50,55]. The high pH coupled with the high concentration of acetyl-CoA in the mitochondrial matrix create a permissive environment for non-enzymatic acetylation of mitochondrial proteins [49,50]. However, these considerations do not exclude the possibility that at least some protein acetylation in the mitochondria is catalyzed by KATs. In addition, some acyl-CoAs, such as 3-hydroxy-3-methylglutaryl-CoA, and glutaryl-CoA, are sufficiently reactive under the in vivo conditions and are able to non-enzymatically modify proteins [56].

## 3. AMPK Regulation of Protein Acetylation

**Figure 1:**
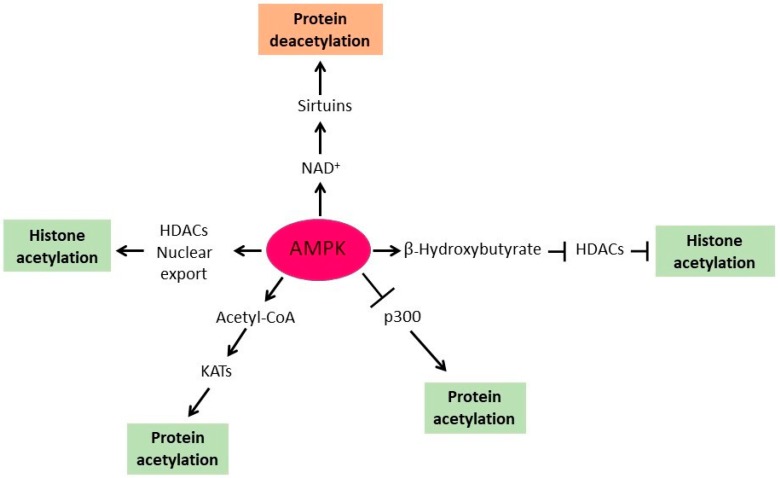
AMP-activated protein kinase (AMPK) regulates protein acetylation by several different mechanisms: (i) AMPK phosphorylates and inhibits acetyl-CoA carboxylase (ACC) and thus elevates acetyl-CoA level and activity of lysine acetyltransferases (KATs); (ii) AMPK increases the cellular concentration of NAD^+^ and thus activates sirtuins; (iii) AMPK upregulates hepatic synthesis of α-hydroxybutyrate, and thus inhibits histone deacetylases (HDACs) and promotes histone acetylation; (iv) AMPK increases histone acetylation in the nucleus by inducing nuclear export of HDACs; (v) AMPK directly phosphorylates and downregulates p300 KAT. Arrows denote activation and t-bars denote inhibition.

AMPK regulates protein acetylation by at least five distinct mechanisms (Figure 1). First, AMPK phosphorylates and inhibits ACC and thus regulates acetyl-CoA homeostasis. Second, AMPK activates sirtuin SIRT1 by increasing the cellular concentration of NAD^+^, a cofactor of sirtuins. Third, AMPK inhibits class I and II histone deacetylases (HDACs) by upregulating hepatic synthesis of α-hydroxybutyrate, a natural inhibitor of HDACs. Fourth, AMPK induces translocation of HDACs 4 and 5 from the nucleus to the cytoplasm and thus increases histone acetylation in the nucleus. Fifth, AMPK directly phosphorylates and downregulates p300 KAT.

### 3.1. Acetyl-CoA Level Regulates Protein Acetylation

Acetyl-CoA is the donor of acetyl groups for protein acetylation and KATs depend on intermediary metabolism for supplying acetyl-CoA in the nucleocytosolic compartment (Figure 1). Acetyl-CoA is thus a key metabolite that links metabolism with signaling, chromatin structure, and transcription [29,30,45,53,57,58,59]. Changing metabolic conditions drive fluctuations of the cellular level of acetyl-CoA to the extent that the activity of KATs is regulated by the availability of acetyl-CoA, resulting in dynamic protein acetylations that regulate a variety of cell functions, including transcription, replication, DNA repair, cell cycle progression, and aging. Acetyl-CoA can freely diffuse through the nuclear pore complex and changes in the pool of available acetyl-CoA in the cytoplasm cause changes in protein acetylation in both the nucleus and cytoplasm. However, the mitochondrial pool of acetyl-CoA is biochemically isolated and cannot be used for histone acetylation in the nucleocytosolic compartment [60]. In mammalian cells, glycolytically produced pyruvate is translocated from the cytosol into mitochondria, where pyruvate dehydrogenase converts it into acetyl-CoA. Acetyl-CoA then enters the tricarboxylic acid (TCA) cycle and condenses with oxaloacetate, producing citrate. Citrate can be subsequently exported from the mitochondrial matrix into the cytosol, where ATP-citrate lyase (ACL) converts it into acetyl-CoA and oxaloacetate. This acetyl-CoA is then used by KATs for protein acetylation in the nucleocytosolic compartment [61], in addition to being a precursor of several anabolic pathways, including de novo synthesis of fatty acids. Since ACL generates acetyl-CoA from glucose-derived citrate, glucose availability affects histone acetylation in an ACL-dependent manner, and when the synthesis of acetyl-CoA is compromised, rapid histone deacetylation ensues [60,61].

Translocation of pyruvate dehydrogenase complex (PDH) from the mitochondria to the nucleus provides an alternative mechanism for synthesis of acetyl-CoA in the nucleus. PDH translocated to the nucleus in a cell-cycle-dependent manner and in response to serum, epidermal growth factor, or mitochondrial stress. Inhibition of nuclear PDH decreased acetylation of specific lysine residues in histones and transcription of genes important for G1-S phase progression [62]. In addition to ACL and nuclear PDH, direct de novo synthesis of acetate from pyruvate for acetyl-CoA production occurs under conditions of nutritional excess [63]. The conversion of pyruvate to acetate takes place either by coupling to reactive oxygen species (ROS) or by the activity of keto acid dehydrogenases, which function under certain conditions as pyruvate decarboxylase [63].

Since nucleocytosolic acetyl-CoA is also used for de novo synthesis of fatty acids, histone acetylation and synthesis of fatty acids compete for the same acetyl-CoA pool. ACC catalyzes the carboxylation of acetyl-CoA to malonyl-CoA, the first and rate-limiting reaction in the de novo synthesis of fatty acids. The ACC activity affects the concentration of nucleocytosolic acetyl-CoA. Attenuated expression of yeast ACC encoded by the *ACC1* gene, increases global acetylation of chromatin histones as well as non-histone proteins, and alters transcriptional regulation [64]. Direct pharmacological inhibition of ACC in human cancer cells also induces histone acetylation [65,66]. ACC is phosphorylated and inhibited by AMPK. In yeast, inactivation of the SNF1 complex, the budding yeast ortholog of mammalian AMPK [67,68,69], results in increased Acc1p activity, reduced pool of cellular acetyl-CoA, and globally decreased histone acetylation [70]. Activation of AMPK with metformin or with the AMP mimetic 5-aminoimidazole-4-carboxamide ribonucleotide (AICAR) increases the inhibitory phosphorylation of ACC, and decreases the conversion of acetyl-CoA to malonyl-CoA, leading to increased protein acetylation and altered gene expression in prostate and ovarian cancer cells [65].

### 3.2. NAD^+^ Synthesis Regulates Protein Acetylation

NAD^+^ is a cofactor used by many oxidoreductases to carry electrons in redox reactions. In addition, NAD^+^ is used as a cosubstrate by a group of HDACs called sirtuins, named after the budding yeast protein Sir2. In mammals, there are seven NAD^+^-dependent sirtuins. Similar to the dependence of KATs on acetyl-CoA level, the activity of sirtuins is regulated by metabolically driven changes in the cellular level of NAD^+^ [71,72]. AMPK activation induces expression of nicotinamide phosphoribosyltransferase (NAMPT), the rate-limiting enzyme in the NAD^+^ salvage pathway that converts nicotinamide to nicotinamide mononucleotide to enable NAD^+^ biosynthesis. AMPK activation thus increases NAD^+^ level, elevating SIRT1 activity [73,74]. SIRT1-mediated protein deacetylation subsequently activates downstream targets, including peroxisome proliferator-activated receptor gamma coactivator 1-α (PGC-1α) and forkhead box protein O1 (FOXO1) [74]. In addition, AMPK directly phosphorylates SIRT1 at T344, which results in dissociation of SIRT1 from the inhibitory bladder cancer protein 1 (DBC1), and SIRT1-mediated deacetylation of p53 and inhibition of its transcriptional activity [75]. Yet another mechanism for SIRT1 activation by AMPK involves phosphorylation of glyceraldehyde 3-phosphate dehydrogenase (GAPDH) by AMPK, leading to nuclear translocation of GAPDH and GAPDH-dependent dissociation of SIRT1 from DBC1 [76].

### 3.3. α-Hydroxybutyrate Synthesis Regulates Protein Acetylation

The ketone body α-hydroxybutyrate is structurally similar to butyrate, an effective inhibitor of class I and II HDACs [77]. Ketone bodies, including α-hydroxybutyrate, are produced during starvation or prolonged exercise, when liver switches the metabolic mode from catabolism of glucose to catabolism of triacylglycerols and fatty acids. This metabolic switch is partly orchestrated by AMPK and activation of AMPK increases fatty acid oxidation, leading to production of ketone bodies, including α-hydroxybutyrate [77]. Administration of exogenous α-hydroxybutyrate or inducing catabolism of fatty acids by calorie restriction increased global histone acetylation in mouse tissues [77]. Acetylation of histones in the promoters of genes required for protection against oxidative stress was also increased, leading to increased expression of the corresponding genes and elevated protection against oxidative stress [77]. These results indicate that AMPK activation induced by calorie restriction or prolonged exercise leads to α-hydroxybutyrate-mediated inhibition of HDACs and globally increased histone acetylation, and may represent one of the health-promoting mechanisms of calorie restriction.

### 3.4. AMPK Induces Nuclear Export of HDACs

Type II HDACs belong into two subgroups, IIa and IIb. HDACs of the IIa subgroup, HDAC4, HDAC5, HDAC7, and HDAC9, are able to shuttle between the nucleus and cytoplasm [78,79,80,81]. AMPK phosphorylates HDAC5 at Ser259 and Ser498, which promotes export of HDAC5 from the nucleus to the cytoplasm. This removal of HDAC5 from the nucleus results in decreased occupancy of HDAC5 and increased histone acetylation at promoters of glucose transporter member 4 (GLUT4), myogenin, and α-catenin genes, leading to increased transcription of the corresponding genes [82,83,84,85,86]. It appears that AMPK regulates expression of host of genes involved in differentiation or development by phosphorylating HDAC5 and HDAC4 and promoting their export from the nucleus. AMPK also mediates nuclear accumulation of transcription factor hypoxia-inducible factor 1α (HIF-1α) by a mechanism that involves HDAC5. Activation of nuclear AMPK promotes nuclear export of HDAC5, presumably by directly phosphorylating HDAC5. Cytosolic HDAC then deacetylates heat shock protein 70 (HSP70), triggering dissociation of HSP70 from HIF-1 and nuclear transport of HIF-1α [84]. Depending on their phosphorylation level, also HDAC7 and HDAC9 shuttle between nucleus and cytoplasm. However, it is not known whether they are AMPK substrates and whether AMPK regulates their nucleocytosolic shuttling.

### 3.5. AMPK Phosphorylates p300 KAT and Histone H3

AMPK directly phosphorylates transcriptional coactivator p300 KAT on Ser89, which inhibits the interaction of p300 with peroxisome proliferator-activated receptor γ (PPAR-γ) and retinoid acid receptor [19]. The AMPK-mediated phosphorylation of p300 also results in decreased acetylation and reduced activity of transcription factors nuclear factor kappa B (NFκB) and SMAD3 [87,88]. AMPK also promotes histone acetylation indirectly through phosphorylation of histone H2B at Ser36, particularly at promoters occupied by p53. During metabolic or genotoxic stress, AMPK translocates to the nucleus, binds to chromatin, and phosphorylates histone H2B at Ser36. This phosphorylation leads to increased assembly and recruitment of KATs to specific promoters, associated with increased transcription [89]. An analogous situation was described also in yeast. SNF1, the yeast AMPK ortholog, phosphorylates histone H3 at Ser10, which results in increased acetylation of Lys14 of histone H3 by KAT Gcn5 in the promoter of the *INO1* gene [90]. It appears that SNF1-mediated phosphorylation of Ser10 of histone H3 does not represent a general mechanism of histone acetylation. Rather, inactivation of SNF1 results in decreased nucleocytosolic level of acetyl-CoA by increasing conversion of acetyl-CoA into malonyl-CoA, which results in globally reduced acetylation of histone and non-histone proteins [70].

## 4. AMPK Is Regulated by Protein Acetylation

**Figure 2:**
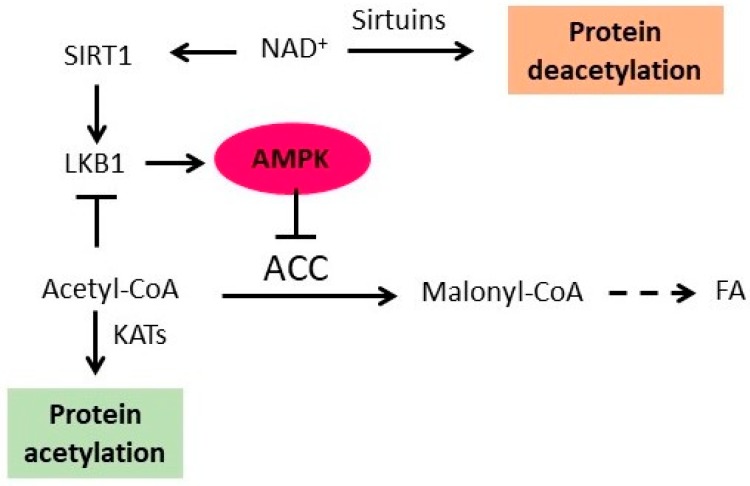
Model of the feedback regulation of AMPK by protein acetylation. AMPK phosphorylates and inhibits ACC, thus increasing acetyl-CoA cellular level and promoting KAT-mediated protein acetylation. Acetylation of liver kinase B1 (LKB1) inhibits the ability of LKB1 to activate AMPK. AMPK also promotes synthesis of NAD^+^, thus activating SIRT1 and other sirtuins and promoting protein deacetylation. SIRT1 deacetylates and activates LKB1, resulting in AMPK activation. Arrows denote activation, t-bars denote inhibition, and dashed arrow indicates multistep pathway.

AMPK activity is regulated by an upstream kinase LKB1, which activates AMPK by phosphorylating it on Thr172. LKB1 is a low energy sensor that regulates tumorigenesis and apoptosis by regulating AMPK and mechanistic target of rapamycin (mTOR) pathways [10]. LKB1 is acetylated and the acetylation reduces its ability to activate AMPK (Figure 2). Deacetylation of LKB1 by SIRT1 activates LKB1 and AMPK and increases inhibitory phosphorylation of ACC [91]. Inhibition of ACC increases acetyl-CoA level and protein acetylation [65] and presumably should also promote LKB1 acetylation and reduced activation of AMPK (Figure 2). It is tempting to speculate that LKB1 acetylation and diminished activation of AMPK form a regulatory loop with ACC, which contributes to regulation of acetyl-CoA homeostasis and protein acetylation [70]. Increased acetyl-CoA level would promote LKB1 acetylation and diminished activation of AMPK. Decreased activity of AMPK would result in lower AMPK-mediated phosphorylation and inhibition of ACC, increased conversion of acetyl-CoA to malonyl-CoA, decreased acetyl-CoA level and decreased protein acetylation. Decreased acetyl-CoA level would also result in hypoacetylation of LKB1 and increased activation of AMPK. This, in turn, would lead to increased phosphorylation and inhibition of ACC, decreased conversion of acetyl-CoA to malonyl-CoA, increased acetyl-CoA level, and increased protein acetylation. In addition, activation of AMPK would promote NAD^+^ synthesis, leading to elevated SIRT1 activity (Figure 2). This homeostatic mechanism would contribute to the regulation of the nucleocytosolic level of acetyl-CoA and NAD^+^ within certain limits and would prevent gross hypoacetylation or hyperacetylation of proteins, a condition that might alter regulation of many essential processes. It also appears that SIRT1 and AMPK mediate the positive effect of some dietary compounds, such as resveratrol and other polyphenols. Resveratrol increases SIRT1 activity, which activates AMPK signaling, presumably by deacetylating LKB1. Activated AMPK then suppresses lipid accumulation in hepatocytes [92].

An analogous regulatory loop seems to operate in yeast. The yeast SNF1 complex consists of the catalytic α subunit Snf1p, one of three different regulatory α subunits, Sip1p, Sip2p, or Gal83p, and the stimulatory γ subunit Snf4p [93]. Acetylation of Sip2p, one of the regulatory α-subunits of the SNF1 complex, results in inhibition of SNF1. The level of Sip2p acetylation depends on the nucleocytosolic level of acetyl-CoA and is increased when *ACC1* transcription is repressed [64] and decreased in *snf1*Δ cells [70]. The acetylation of Sip2p increases its interaction and inhibition of Snf1p [94]. This results in activation of the *ACC1* gene and reduced cellular level of acetyl-CoA, which promotes deacetylation of Sip2p and activation of SNF1. Thus, in both yeast and mammalian cells, AMPK/SNF1 regulate protein acetylation and are themselves regulated by protein acetylation. Since SNF1 also phosphorylates Sch9, a yeast ortholog of the Akt kinase, inhibition of SNF1 by Sip2p results in reduced phosphorylation of Sch9, ultimately leading to extended life span. Acetylation of Sip2p thus promotes life span extension and Sip2p acetylation mimetics are more resistant to oxidative stress [94].

## 5. Conclusions

AMPK is an energy sensor and master regulator of metabolism, functioning as a fuel gauge. AMPK phosphorylates and regulates a number of metabolic enzymes and transporters, as well as transcription factors. In this review article, we have focused on the role of AMPK in regulation of protein acetylation and on regulation of AMPK by protein acetylation. Taken together, AMPK regulates protein acetylation by regulating synthesis of acetyl-CoA, NAD^+^, and α-hydroxybutyrate, as well as by directly phosphorylating and regulating KATs and HDACs. Using these two general mechanisms, AMPK contributes to the global regulation of protein acetylation and connects epigenetic chromatin modifications with the cellular metabolic state. Since protein acetylation appears to be as widespread as protein phosphorylation, it endows AMPK with yet another mechanism of regulation of cellular and organismal physiology.
